# Radiation Dose and Image Quality of a High-Pitch Prospective Spiral First Approach in Coronary Computed Tomography Angiography (CCTA)

**DOI:** 10.3390/jcdd8100119

**Published:** 2021-09-24

**Authors:** Tom Finck, Konstantin Klambauer, Eva Hendrich, Albrecht Will, Stefan Martinoff, Martin Hadamitzky

**Affiliations:** Institute of Radiology and Nuclear Medicine, German Heart Centre Munich, Lazarettstr. 36, 80636 Munich, Germany; Tom.Finck@mri.tum.de (T.F.); k.klambauer@campus.lmu.de (K.K.); hendrich@dhm.mhn.de (E.H.); will@dhm.mhn.de (A.W.); drmartinoff@dhm.mhn.de (S.M.)

**Keywords:** coronary computed tomography angiography, high-pitch-spiral scan, radiation exposure, coronary artery disease

## Abstract

**Objective:** To investigate a high-pitch spiral first (HPSF) approach for coronary computed tomography angiography (CCTA) in an unselected patient cohort and compare diagnostic yield and radiation exposure to CCTAs acquired via conventional, non-high-pitch spiral first (NHPSF) scan regimes. **Materials and Methods:** All consecutive patients from 1 January 2015 to 31 December 2017 were included. Two investigation protocols (HPSF/NHPSF) were used with the aim to achieve diagnostic image quality of all coronary segments. Low-pitch secondary scans followed the initial examination if image quality was unsatisfactory. Dosage and image quality were compared between both regimes. **Results:** 1410 patients were subject to a HPSF and 236 patients to a NHPSF approach. While the HPSF approach led to a higher fraction of re-scans (35% vs. 11%, *p* < 0.001), the fraction of aggregate scans that remained non-diagnostic after considering the initial and secondary scan was comparably low for the HPSF and NHPSF approach (0.78 vs. 0%, *p* = 0.18). Aggregate radiation exposure in the HPSF protocol was significantly lower (1.12 mSv (IQR: 0.73, 2.10) vs. 3.96 mSv (IQR: 2.23, 8.33) *p* < 0.001). **Conclusions:** In spite of a higher number of re-scans, a HPSF approach leads to a reduction in overall radiation exposure with diagnostic yields similar to a NHPSF approach.

## 1. Introduction

The excellent negative predictive value of coronary computed tomography angiography (CCTA) has put it in the spotlight as a non-invasive test for coronary artery disease (CAD). More widespread implementation of this method underlines the importance to continuously reduce radiation to exposed patients, a quest in part resolved by a combination of hardware adaptions and prospective ECG-gating [1,2].

While the improvements in dose-reduction are certainly paramount, one should nonetheless stress that clinical decision making based on CCTA results calls for reliable image quality and that a non-diagnostic scan may lead to further radiation exposure by alternative modalities such as invasive coronary angiography or Sesta-MIBI myocardial perfusion scan. In this setting, the potentially artefact-compromised data in high-pitch scans especially in patients with extrasystoles or elevated heart rates has often raised concerns about its robustness in a broad patient collective. One has to note, that CCTA can only be a reliable go-to method for non-invasive cardiac imaging if the coronary tree can be evaluated with diagnostic certainty. Ideally, such is the case after the first scan. Otherwise re-scans are necessary until the aggregate interpretation of all acquired data allows for the valid interpretation of each coronary segment. Rapidly moving coronary arteries make it difficult to achieve this goal; a common concept to improve image quality is to widen the acquisition window in order to allow for multiple reconstructions at different timepoints of the heart cycle. This of course increases radiation exposure. With the particularly low radiation exposure of the high pitch spiral scan another strategy of acquiring a low dose scan at a single timepoint first and repeating the scan in case of limited image quality is feasible. As long as the fraction of repeat scans is low, this strategy would translate to an overall radiation dose reduction.

The PROTECTION IV study performed a groundwork multicenter exploration of high-pitch spiral CCTA, demonstrating the robust image quality and significant dose reduction when using this technique. Yet, inclusion was restricted to patients with a low and stable heart rate rendering assumptions of a high-pitch approach in an unselected patient cohort highly speculative [3].

To clarify the potential of high-pitch scanning as a go-to method in a broader patient collective, i.e., including those with elevated heart rates and extrasystoles, we investigated radiation exposure and image quality among two examination algorithms that ultimately aimed to achieve CCTAs of diagnostic quality for each patient, namely a high-pitch spiral first (HPSF) approach and non-high-pitch spiral first (NHPSF) approach.

## 2. Materials and Methods

### 2.1. Inclusion/Exclusion

Eligible for retrospective analysis were all consecutive patients referred for CCTA at our institution from 1 January 2015 to 31 December 2017. To investigate cardiac-specific radiation exposure, all scans extending beyond the aortic root or diaphragm were excluded. As patients presenting with atrial fibrillation are routinely not investigated via high-pitch spiral scan at our institution, these were not eligible for comparative analysis and excluded. Informed consent was received before scan acquisition.

### 2.2. General Scan Protocol

All patients were investigated using a dual-source Siemens SOMATOM FORCE (Siemens Healthineers, Forchheim, Germany) scanner. Scan preparation was done by administering up to four doses of 5 mg metoprolol i.v. in patients with a heart rate >60 beats per minute (bpm) as well as 0.8 mg of nitroglycerin sublingually for patients with a systolic blood pressure of at least 100 mmHg. An initial topogram was acquired, the scan area was defined as between the level of the carina and diaphragmatic surface of the heart. After a bolus timing single-slice scan, contrast application (Iomeprol, Imeron 350, Bracco Altana Pharma GmbH, Konstanz, Germany, iodine content 350 mg/cc) followed by a 50 cc saline chaser was performed at an injection rate of 4–6 cc/s for CCTA. Tube voltage and tube current were determined automatically based on the body habitus of the patient (CareDose 4D and CareKV, Siemens Healthineers, Forchheim, Germany). CT acquisition parameters were as follows: slice collimation 3 mm × 192 mm × 0.6 mm, pitch of 3.4. ECG-gated reconstruction algorithms led to a temporal resolution up to 66 ms. For analysis, multiplanar reconstructions (MPR), maximum intensity projections (MIP) and 3D volume rendered (VR) techniques were used. Heart rate and presence of sinus rhythm or extrasystoles was documented during the acquisition.

### 2.3. Scan Methods and Acquisition Algorithm

Two investigation protocols were used in this study with the aim to ultimately achieve diagnostic image quality of all coronary segments. The HPSF protocol consisted of a prospectively ECG-triggered high-pitch spiral CCTA in mid-diastole, followed by a low-pitch method (prospective step-and-shoot (SAS) scan, spiral scan with retrospective gating) or prospective high-pitch spiral with double acquisition in the case of non-diagnostic initial image quality. The NHPSF approach firstly used SAS, retrospectively gated spiral scans or prospective high-pitch scans with double acquisition, followed by a second low-pitch scan method if image quality was deemed unsatisfactory. Repeat scanning was performed in both protocols either holo-cardiac or segment-specific depending on which segments of the coronary tree were initially non-diagnostic. For calcium scoring a low dose native acquisition was performed prior to the selected protocol on every patient. The choice of scan method was nonrandomized at the discretion of the examining senior radiologist depending on patient characteristics, such as heart rate, weight, extrasystoles and pre-existent patient conditions. If obstructive CAD was diagnosed on the first scan, no repeat imaging was performed even if other coronary segments were of non-diagnostic quality and patients were immediately referred for further work-up.

### 2.4. Image Analysis

Each coronary segment was evaluated for the presence of plaques, defined as any structure within and/or adjacent to the coronary artery lumen >1 mm that could be clearly distinguished from the vessel lumen [4]. Images were evaluated on a per-patient and per-vessel basis.

Segmental evaluation of coronary arteries was performed on the basis of the classification of the American Heart Association, using the first 15 of the proposed coronary segments [5].

**Figure 1:**
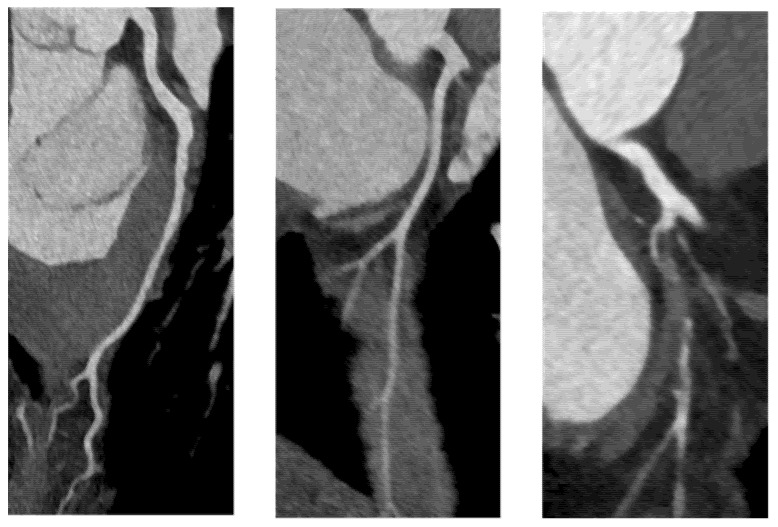
Rating score for the image quality of coronary arteries using curved planar reformations. From left to right: grade 2—excellent (good interpretability in all coronary segments); grade 1—diagnostic (reduced interpretability of at least one coronary segment); grade 0—non-diagnostic (at least one coronary segment is not interpretable).

Image quality of each coronary segment was retrospectively analyzed by one examiner to reduce interobserver variability and the following rating score was used for evaluation (illustrated in Figure 1):

- Grade 0: Non-diagnostic scan, need for repetition
- Grade 1: Diagnostic scan, mild artefacts, good to satisfactory contrast
- Grade 2: Excellent scan, no artefacts, clear contrast in all coronary segments

On a per-patient basis, the lowest score of all coronary segments was used as rating. In coronary segments with a non-diagnostic grade 0 rating, the reasons for poor image quality were further specified (i.e., motion artefacts, insufficient contrast, blooming artefacts due to calcification or scan repetition due to an unrecognizable intramyocardial vessel course). Mean ratings for the first and, if performed, second scan were calculated for the HPSF and NHPSF cohorts.

### 2.5. Radiation Exposure

The effective patient dose was calculated as proposed by the European Working Group for Guidelines on Quality Criteria in CT: effective radiation dose (mSv) is the product of dose-length product (DLP) and a thorax-specific conversion factor [effective dose = k × DLP (mGy/cm)]. The CT volume dose index (CTDI_vol_) and DLP values were obtained from the scan protocol. In accordance to accepted guidelines, a chest-specific conversion factor (k) of 0.014 mGy^−1^ cm^−1^ was used for effective dose calculation [6].

### 2.6. Statistical Analysis

Categorical variables are expressed as frequencies and percentages. Continuous variables are described as means ± standard deviation or as median and interquartile range in the case of non-normal distribution. Student’s *t*-test as well as Mann–Whitney U were used to test for differences in continuous variables and chi-square was used for categorical values. All statistical tests were performed two-sided at a significance level of 5%. The statistical package R (R Core Team, Vienna, Austria) version 2.10.1 including the package rms was used for statistical analysis [7].

## 3. Results

### 3.1. Patients

In total, 1797 patients were investigated via CCTA during the study period. Of these, 121 were excluded because the scan extended beyond the cardiac region. Thirty patients were investigated with a double high pitch spiral scan on the ground of atrial fibrillation and therefore excluded. The study population of 1646 patients consisted of 1410 patients with a HPSF and 236 patients with a NHPSF approach.

Due to the nonrandomized nature of this study, there were significant differences in patient characteristics of the HPSF and NHPSF cohort, including a higher heart rate, BMI and more frequent intra-procedural recordings of extrasystoles for the latter. Furthermore, due to the “allcomer” design, there was some missing patient data concerning heart rate, heart rhythm and BMI. Patients with missing data were subsequently excluded from subgroup analysis (Appendix A). Detailed patient characteristics are provided in Table 1.

### 3.2. Image Quality

**Figure 2:**
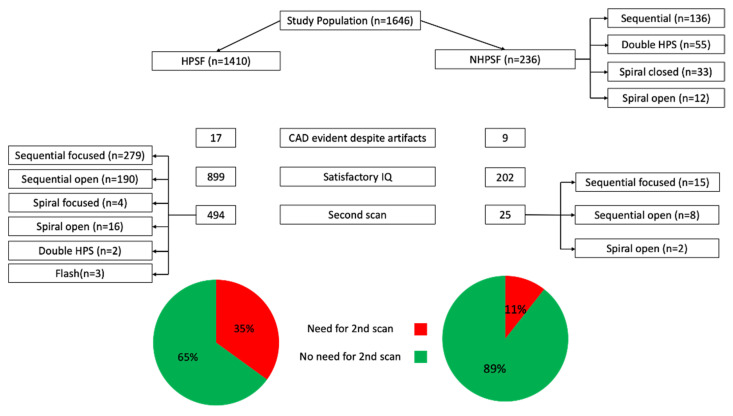
Flow chart of image acquisition. RS: retrospective spiral, SAS: prospective step-and-shoot, HPS: high-pitch spiral; HPSF: high-pitch spiral first; NHPSF: non-high-pitch spiral first; IQ: image quality; CAD: coronary artery disease.

In the 1410 patients with a HPSF protocol, analysis on a per-patient basis revealed that image quality in the first scan was excellent or diagnostic in 899 (63.8%) patients, compared to 202 (85.6%) patients with a NHPSF approach (*p* < 0.001). In detail, significantly more patients in the HPSF-group had an excellent (34.6% vs. 23.2%, *p* < 0.001) but also non-diagnostic (36.2% vs. 14.4%, *p* < 0.001) image quality on initial scanning. In total, 17 patients in the HPSF-group and 9 patients in the NHPSF-group had initial scans with non-diagnostic quality of some coronary segments but did not get repeat scans due to a relevant stenosis that was evident in the diagnostic coronary segments. A flow-chart illustrating the methods of scan acquisitions is given in Figure 2.

**Figure 3:**
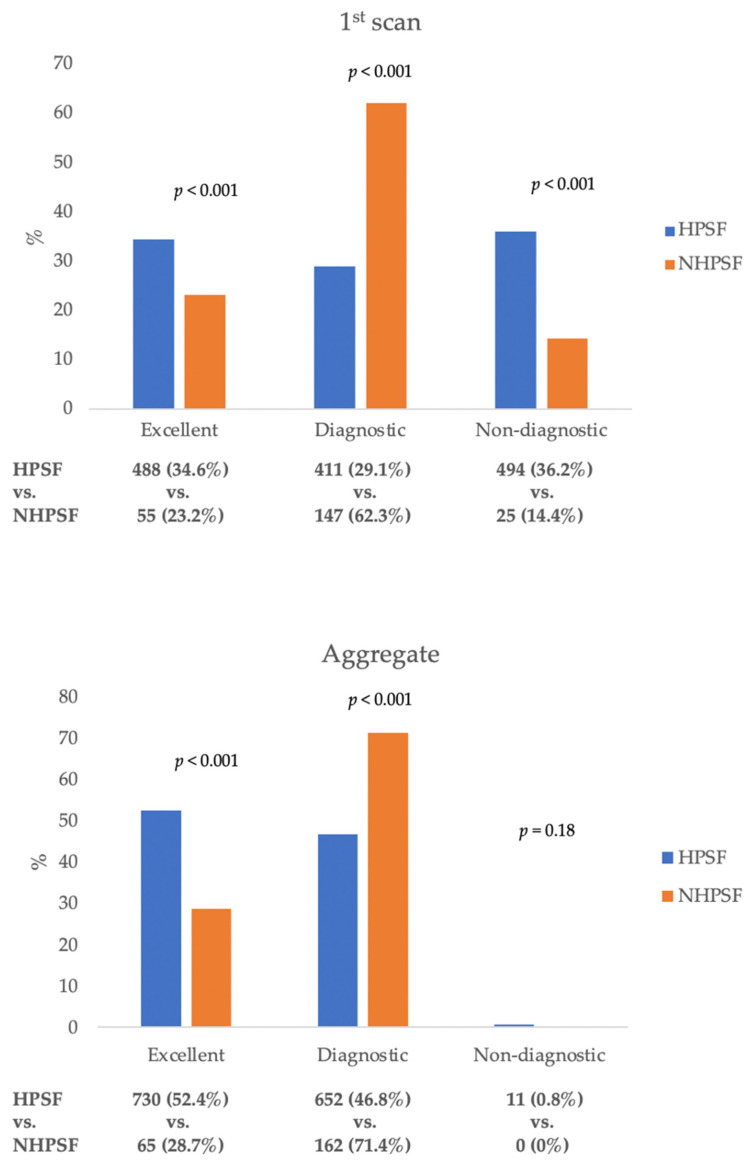
The image quality of the first scan as well as the aggregate of the first and potential second CCTA for patients with the HPSF and NHPSF protocol. HPSF: high-pitch spiral first; NHPSF: non-high-pitch spiral first.

Figure 3 illustrates the image quality of the respective first and aggregate scans. The count of examinations that remained non-diagnostic after repeated scanning was comparable for the NHPSF-protocol and HPSF-protocol while the number of excellent scans on aggregate analysis was significantly greater in patients getting the HPSF protocol.

**Figure 4:**
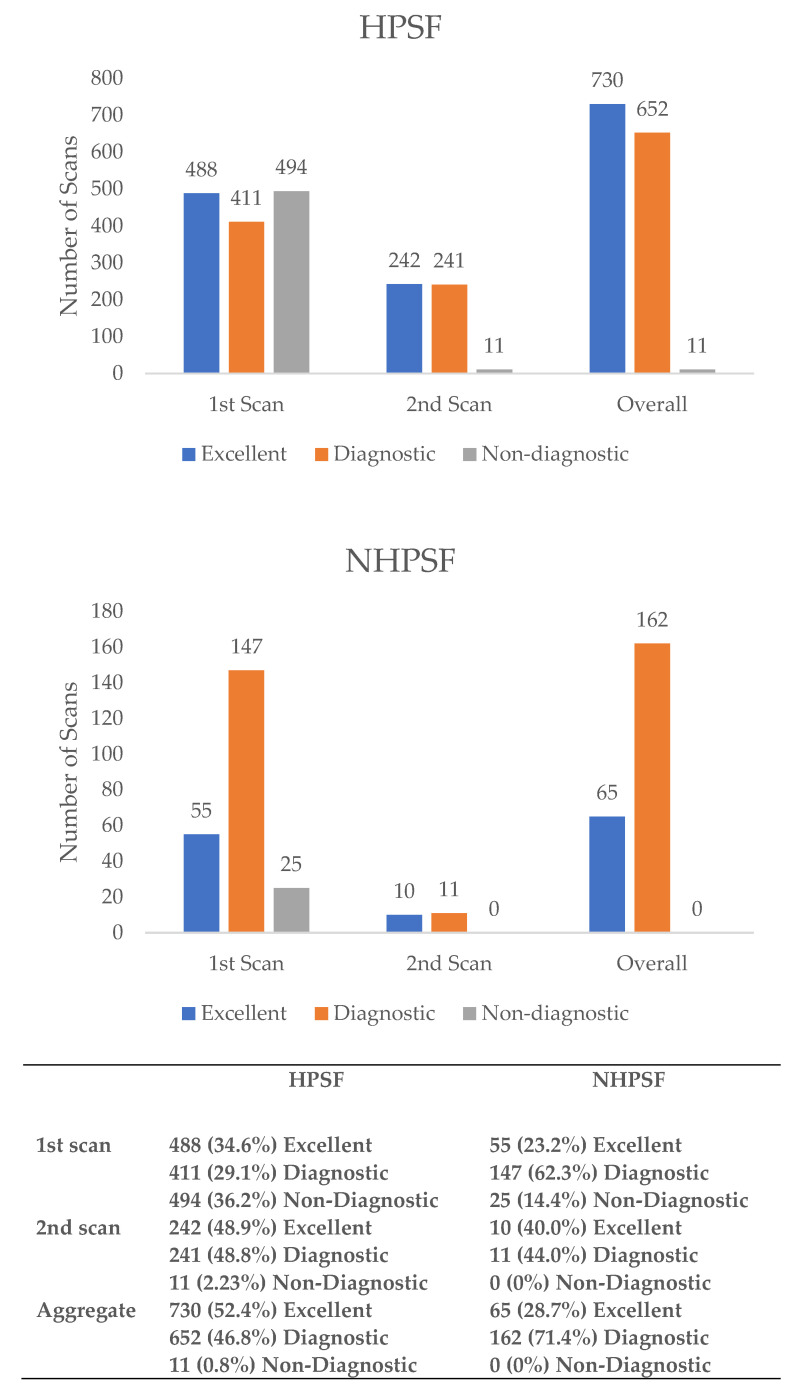
Image quality of the first and repeat scans for patients with a HPSF and NHPSF approach. HPSF: high-pitch spiral first; NHPSF: non-high-pitch spiral first.

Figure 4 further specifies how and in what quality initially non-diagnostic scans were repeated. The percentage of aggregate scans of excellent quality was significantly higher in the HPSF group compared to the NHPSF group (*p* < 0.001).

**Figure 5:**
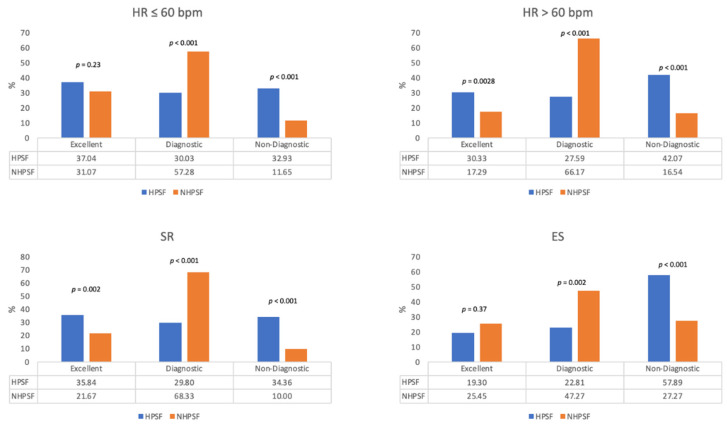
The scan quality of the first scan for patients investigated with the HPSF or NHPFS protocol after dichotomization for heart rate or cardiac rhythm. HPSF: high-pitch spiral first; NHPSF: non high pitch spiral first; SR: sinus rhythm without extrasystoles; ES: extrasystoles; bpm: beats per minute.

Both protocols allowed for similar fractions of excellent initial scans in patients with heart rates below 60 bpm. Higher rates of excellent image quality could yet be obtained when using the HPSF vs. NHPSF approach in patients with sinus rhythm and no extrasystoles during scan acquisition. This advantage faded however if extrasystoles occurred as both protocols then had similar fractions of excellent scans and the HPSF protocol led to more scans of non-diagnostic quality. Further, the occurrence of primary successful CCTAs was significantly reduced in patients examined with the HPSF approach if heart rates were beyond 60 bpm. Refer to Figure 5 and Table 2 for rates of primary successful scans as a function of clinical and demographical parameters.

### 3.3. Artefact Distribution

Taken together, 1976, respectively 100 coronary arteries were characterized for artefacts in the 494 HPSF and 25 NHPSF patients that needed repeat scans (LM, LAD, LCX and RCA in each patient). There was a trend for more artefact-ridden coronary arteries in the NHPSF than HPSF group mainly due to a higher rate of coronary arteries that were affected by motion artefacts. Artefact analysis for non-diagnostic scans is given in Table 3.

### 3.4. Radiation Exposure

**Figure 6:**
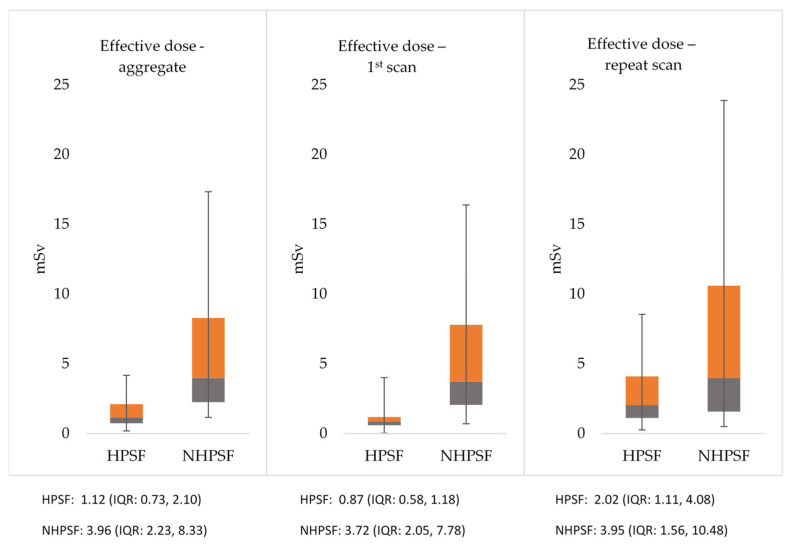
Radiation exposure (median, interquartile-range) for patients investigated with a HPSF and NHPSF approach. HPSF: high-pitch spiral first; NHPSF: non high pitch spiral first. All results are significant with a *p*-value < 0.001.

The HPSF-cohort had a significantly lower radiation exposure than the NHPSF-cohort for the first scan. In the 494 (HPSF-cohort) and 25 patients (NHPSF-cohort) with repeat scans, the effective dose was also lower if HSPF was used. Hence, on aggregate, a significantly lower overall radiation exposure was noted for the HPSF-protocol. Detailed results on radiation exposure are given in Figure 6.

**Figure 7:**
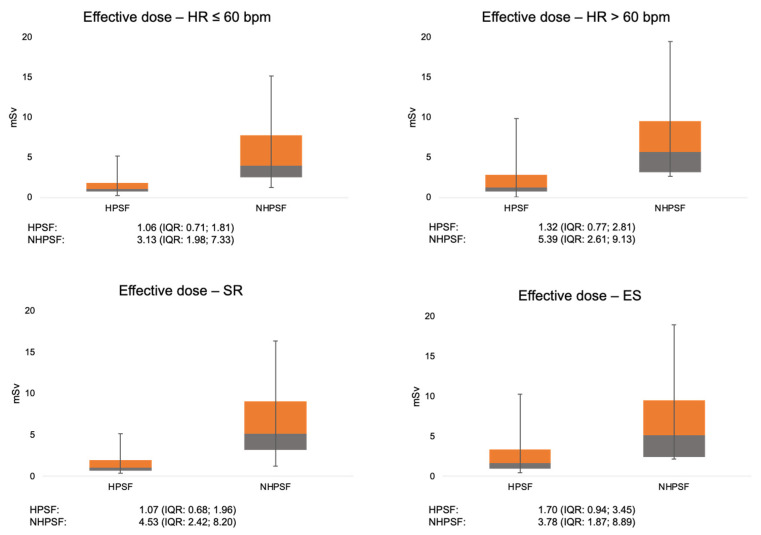
Radiation exposure (median, interquartile-range) for patients investigated with a HPSF and NHPSF approach after dichotomization for heart rate and extrasystoles. HPSF: high-pitch spiral first; NHPSF: non high pitch spiral first; SR: Scheme 0.

Dichotomization for heart rate revealed that the HPSF approach had significantly lower radiation doses for both, patients with heart rates below and above 60 bpm. Similarly, utilizing the HPSF approach reduced radiation exposure irrespective if patients had extrasystoles or not (Figure 7). Subgroup analysis of radiation exposure as a function of clinical and demographical parameters is given in Table 4.

In patients with elevated BMI (≥25), a marked increase in radiation exposure could be noted for both, the HPSF and NHPSF protocol. On the other hand, rise of overall radiation exposure as a function of elevated heart rates (>60 bpm) or lacking extrasystole-free sinus rhythm could be noted in the HPSF approach only.

## 4. Discussion

In an effort to elucidate the potential of high-pitch prospective CCTA acquisition in a broad patient collective, our study suggests that (i) the implementation of a HPSF approach is inching towards sub-millisievert scanning and can significantly reduce radiation exposure and (ii) albeit a higher rate of repeat scans is necessary in a HPSF-concept, the amount of scans that remain non-diagnostic after repeat scanning is not significantly different from a conventional CCTA approach.

X-ray based medical imaging is consistently challenged by an effort to lower radiation exposure while maintaining or even improving diagnostic quality.

Multicenter evaluation of the dose-saving potential of high-pitch spiral vs. conventional scan methods has demonstrated the non-inferior image quality, impressive dose reduction and more frequent re-scanning in selected patients [3]. While the higher rate of initially non-diagnostic scans in the underlying study (35% vs. 14%) might be linked to lack of heart-rate restriction in our cohort, evolutions in CT hardware and consecutive acceleration of scan velocities help explain the more pronounced dose reduction (72% vs. 58%) for HPSF in the here-presented data.

Noteworthy works in this domain also reported low radiation exposures of around 1.3 mSv and good, yet heart-rate dependent image quality in 256 patients investigated with this method [2]. This is compatible with our data as the fraction of patients with excellent high-pitch spiral scans in the underlying study decreased by around 20% if heart rates were above 60 bpm but nevertheless remained significantly higher than if a NHPSF approach was used.

Another working group was able to report comparable image quality with notably lower radiation exposure (0.53 ± 0.14 vs. 1.33 ± 0.17 mSv) in patients scanned with a high-pitch spiral compared to lower-pitch sequential scans [8].

Further studies regarding other aspects of high-pitch spiral CCTA, be it its applicability for free-breathing acquisition, the rate of scans that were of non-diagnostic quality, or its robust image quality after tube voltage reduction, confirm the actuality of this matter [9,10,11]. Reports about the routine use of high-pitch spiral CCTA in an every-day clinical setting are nonetheless scarce. While groundwork studies have in the past elucidated the potential of high-pitch spiral CCTA to reduce radiation dose without compromising image quality, these studies have all been carried out in pre-selected patient cohorts of notably smaller size [2,3,12,13,14]. Ultimately, our study is the first to investigate the practicability of such a regime in a broader patient population not restricted to individuals with heart rates below 60 bpm and extrasystole-free sinus-rhythm.

Up to date, one area of concern has been that beyond a certain detector pitch, susceptibility to artefacts rises exponentially, potentially leading to a higher rate of repeat scanning. This is confirmed by our observation of a significantly higher rate of re-scans in the HPSF approach, affecting around one-third of the patients. Previous studies reported notably lower rates of non-diagnostic high-pitch spiral scans, a discrepancy potentially explained by the broad patient cohort in the underlying study [2,12,15,16]. Susceptibility of high-pitch spiral acquisitions to motion artefacts nonetheless remains an area of concern that cannot even be overcome with the most sophisticated iterative reconstruction techniques [17,18]. Hence, it comes to no surprise that elevated heart rates or extrasystoles went along with a substantial drop in primary successful HPSF-examinations as the prospective high-pitch nature of image acquisition only leaves little room for scan reconstruction in an alternative phase of the R-R interval [19].

In the vast majority of initially non-diagnostic cases only one or a few coronary segments needed repeat imaging and therefore the repeat scan only partly covered the coronary tree. Hence, the aggregate ED was around two-thirds lower if a high-pitch spiral was used ad initio. In detail, the “scan-to-diagnosis” ED was 1.12 vs. 3.96 mSv in favor of the HPSF protocol. In spite of the notable rise in ED, that difference remained significant when analyzing subpopulations with extrasystoles or elevated heart rates. Furthermore, and in agreement to established data, a higher BMI went in-hand with a rise in radiation exposure as dose modulation techniques counterbalanced the expected fall in contrast-to-noise [20]. It needs to be said that the higher rate of repeat scans inevitably called for repeat contrast injections, a fact that could be of concern in patients with impaired renal function or thyroid disease.

The here-stated recommendation for HPSF as a go-to approach in CCTA holds true especially in patients with heart rates below 60 bpm and lacking extrasystoles. While we provide data on reduced radiation exposure in patients with higher heart rates or extrasystoles, more evidence in this regard is needed to conclusively validate the utility of a HPSF approach.

Concerns on the robustness of HPSF imaging can be mitigated by our finding that the image quality of this approach is comparable to conventional scan regimes, in-line with results from other working groups that validated the image quality of high-pitch spiral scans [14,21,22,23]. Indeed, the diagnostic yield of CCTAs in the HPSF approach was comparable to a NHPSF approach, allowing 1399 of 1410 patients to benefit from a non-invasive, low-radiation coronary imaging with comparable negative predictive value to invasive coronary angiography [24,25].

A limitation of this study is the observational, nonrandomized design. Hence, decisions regarding the scan protocol were taken by the investigator and presumably taken conservatively if difficult examination conditions were expected. Consequently, the NHPSF-cohort presented with higher heart rates, more extrasystoles and a higher BMI, all known factors to impair the image quality of a CCTA. Hence, a selection bias of the chosen scan protocol might have contributed to the superior image quality in the HPSF cohort. Furthermore, the NHPSF cohort had higher calcium score levels and a higher per-patient number of calcified plaques as patients with more protruded coronary artery disease were less likely to have sinus rhythm and might therefore have been more readily examined with a NHPSF approach.

## 5. Conclusions

This study demonstrates that a HPSF approach for CCTA in an unselected patient collective allows for significant reductions in radiation exposure with similar diagnostic yields to conventional CCTA protocols.

## Figures and Tables

**Table 1:** Patient characteristics. HPSF: high-pitch spiral scan first; NHPSF: non high-pitch spiral scan first; HR: heart rate; SR: sinus rhythm; ES: extrasystoles; BMI: body mass index; bpm: beats per minute.

	HPSF (n = 1410)	NHPSF (n = 236)	*p*
HR (bpm)	58 ± 12 (n = 1273)	63 ± 13 (n = 203)	<0.001
Male (%)	64.6	71.6	0.036
BMI	25.8 ± 3.4 (n = 1258)	27.2 ± 4.8 (n = 208)	<0.001
SR	943 (88.6%) (n = 1064)	120 (68.6%) (n = 175)	<0.001
ES	121 (11.4%) (n = 1064)	55 (31.4%) (n = 175)	<0.001
Calcium score (Agatston units)	73 ± 159	326 ± 331	<0.001
Contrast agent aggregate (cc)	78 ± 34	77 ± 27	0.67
Metoprolol dose (mg)	5 ± 6	6 ± 6	0.018
Calcified plaques	3 ± 5	9 ± 8	<0.001
Scan length (mm)	126.4 ± 15	122.4 ± 15	<0.001
Tube voltage (kVp)	91.6 ± 13	101.6 ± 16	<0.001

**Table 2:** Subgroup analysis for primary successful (i.e., at least diagnostic image quality of all coronary segments) scans as a function of selected clinical and demographical parameters. The rate of primary successful scans with HPSF was significantly lower than with NHPSF in all subgroups (*p* < 0.05). HPSF: high-pitch spiral scan first; NHPSF: non high-pitch spiral scan first; HR: heart rate; SR: sinus rhythm without extrasystoles; ES: extrasystoles; BMI: body mass index; bpm: beats per minute.

Primary Successful	HPSF	*p*	NHPSF	*p*
	Successful 1st Scans		Successful 1st Scans	
HR ≤ 60 bpm (n = 1003)	612/900 = 68.0%	0.003	93/103 = 90.3%	0.34
HR > 60 bpm (n = 437)	221/373 = 59.2%		86/100 = 86.0%	
SR (n = 1063)	629/943 = 66.7%	<0.001	112/120 = 93.3%	0.004
ES (n = 176)	51/121 = 42.1%		43/55 = 78.2%	
Male (n = 1079)	585/910 = 64.3%	0.47	151/169 = 89.3%	0.79
Female (n = 567)	331/500 = 66.2%		59/67 = 88.1%	
BMI < 25 (n = 645)	387/573 = 67.5%	0.017	62/72 = 86.1%	0.44
BMI ≥ 25 (n = 831)	424/695 = 61.0%		122/136 = 89.7%	

**Table 3:** Artefact analysis for the 1976 and 100 coronary arteries investigated in the 494, respectively 25 patients with non-diagnostic initial scans. HPSF: high-pitch spiral scan first; NHPSF: non-high-pitch spiral scan first. LM: left main coronary artery; LAD: left anterior descending coronary artery; LCX: left circumflex coronary artery; RCA: right coronary artery; intramyocardial: a segment of the coronary artery is surrounded by myocardial tissue by >50% of the circumference.

	HPSF (n = 1976)	NHPSF (n = 100)	*p*	LM	LAD	LCX	RCA	Total
Motion	441 (22.3%)	39 (39.0%)	<0.001	34	108	104	234	480
Calcification	216 (10.9%)	5 (5.0%)	0.06	10	159	36	16	221
Insufficient contrast	16 (0.8%)	0	0.37	0	6	6	5	17
Image noise	19 (1.0%)	1 (1.0%)	>0.99	2	5	6	6	19
Intramyocardial	10 (0.5%)	0	0.48	0	10	0	0	10
Total	702 (35.5%)	45 (45.0%)	0.054	46	288	152	261	747

**Table 4:** Subgroup analysis of overall radiation exposure (mSv) as a function of selected clinical and demographical parameters. Radiation exposure with HPSF was significantly lower than with NHPSF in all subgroups (*p* < 0.05). HPSF: high-pitch spiral scan first; NHPSF: non high-pitch spiral scan first; HR: heart rate; SR: sinus rhythm without extrasystoles; ES: extrasystoles; BMI: body mass index; bpm: beats per minute.

Radiation Exposure	HPSF	*p*	NHPSF	*p*
	mSv		mSv	
HR ≤ 60 bpm (n = 1003)	1.06 (0.71; 1.81)	0.001	3.13 (1.98; 7.33)	0.015
HR > 60 bpm (n = 437)	1.32 (0.77; 2.81)		5.39 (2.61; 9.13)	
SR (n = 1063)	1.07 (0.68; 1.96)	<0.001	4.53 (2.42; 8.20)	0.55
ES (n = 176)	1.70 (0.94; 3.45)		3.78 (1.87; 8.89)	
Male (n = 1079)	1.22 (0.83; 2.28)	<0.001	4.36 (2.33; 8.49)	0.21
Female (n = 567)	0.95 (0.57; 1.81)		3.37 (1.90; 8.00)	
BMI < 25 (n = 645)	0.78 (0.54; 1.42)	<0.001	2.37 (1.39; 4.34)	<0.001
BMI ≥ 25 (n = 831)	1.46 (0.96; 3.09)		5.59 (2.84; 10.02)	

## Data Availability

The data presented in this study are available on request from the corresponding author. The data are not publicly available due to national data security laws.

## Appendix A

Subgroup analysis were performed excluding patients with missing data. This concerned calculations in Table 1, Table 2 and Table 4 as well as Figure 5 and Figure 7:

Data on heart rate was not available for 137 (subgroup: n = 1273) patients in the HPSF group and 33 (subgroup: n = 203) patients in the NHPSF group; Data on BMI was not available for 152 (subgroup: n = 1258) patients in the HPSF group and 28 (subgroup: n = 208) patients in the NHPSF group; Data on heart rhythm (SR/ES) was not available for 346 (subgroup: n = 1064) patients in the HPSF group and 61 (subgroup: n = 175) patients in the NHPSF group.
